# Detection and Early Detection of Pathological Motor Evoked Potentials: A Novel Approach for Intraoperative Neuromonitoring Assistance

**DOI:** 10.1007/s41666-026-00245-9

**Published:** 2026-07-10

**Authors:** Alberto Azzari, Alessandro Boaro, Federica Basaldella, Sonia Nunes, Francesco Sala, Carlo Combi, Pietro Sala, Manuele Bicego

**Affiliations:** 1 Department of Computer Science, University of Verona, Verona, 37134 VR Italy; 2 Section of Neurosurgery, Department of Neurosciences, Biomedicine and Movement Sciences, University of Verona, Verona, 37134 VR Italy; 3 Division of Neurology, University Hospital at the University of Verona, Verona, 37134 VR Italy

**Keywords:** Isolation Forest, Ensemble Learning, Intraoperative monitoring, Surgery, Neurosurgery

## Abstract

Intraoperative monitoring of motor evoked potentials (MEPs) plays a crucial role in detecting potential neurological complications during surgical procedures. Traditional methods rely on real-time human interpretation, which can introduce delays or, worse, errors in response. This study proposes an early detection framework for pathological MEPs, leveraging an anomaly detection approach based on an extension of isolation forests–one of the most effective anomaly detection models. By modeling the temporal evolution of MEPs, our approach not only identifies pathological deviations but also anticipates their occurrence, enabling proactive intervention. We validated our methodology on a dataset of intraoperative MEP recordings, demonstrating improved detection performance and enhanced early warning capabilities compared to conventional approaches. The findings suggest that this framework may support real-time intraoperative monitoring and serve as a foundation for future decision-support tools, although prospective clinical validation is still required before any deployment can be considered.

## Introduction

Intraoperative monitoring (IOM) of motor evoked potentials (MEPs) enables real-time assessment of the functional integrity of motor pathways. It has become a valuable tool in neurosurgery, orthopedics, and vascular surgery procedures [1, 2]. Minimizing the risk of disabling motor deficits is of primary importance during these surgeries. However, achieving optimal outcomes without compromising the patient’s functional status presents a major challenge. MEP alterations should serve as critical warning signs, prompting the surgical team to intervene or pause to prevent irreversible injury to the nervous system. Established warning criteria include MEP disappearance, amplitude reduction, threshold elevation, and morphology simplification [3]. Unlike D waves, which have stable properties and limited clinical indications, muscle MEPs are widely used but inherently polysynaptic, nonlinear, and highly variable. This variability complicates the establishment of robust warning criteria and makes MEPs unusually sensitive to intraoperative physiological changes [4]. While there is consensus on the clinical interpretation of MEP signal alterations, cut-off values, such as a reduction in amplitude greater than 50% during brain surgery or 80% during spinal cord surgery, are often derived empirically, leading to potential inconsistencies in clinical decision-making [5].

Recent advancements in biomedical data analysis, particularly in machine learning and deep learning, have demonstrated their potential to extract and summarize clinically meaningful information across medical imaging, histology data, and biological signals [6–8]. Specifically, several machine learning techniques have been successfully applied to classify cardiac arrhythmias from electrocardiograms (EKG), detect seizures from electroencephalograms (EEG), analyze muscular activity from surface electromyography (sEMG) for robotic prosthesis control, and recently, for the accurate characterization of MEPs based on the muscles that generate them [9–13]. These approaches, when adapted to intraoperative MEP analysis, could refine current warning criteria by unveiling new, actionable signal features, ultimately strengthening their predictive and diagnostic value [14].

One of the key unexplored challenges in intraoperative neurophysiology is the real-time detection of anomalous motor evoked potentials, which plays a crucial role in surgical guidance. Identifying pathological MEP patterns can provide immediate warnings to the surgeon, allowing for timely intervention when abnormal events occur. Unlike traditional time series anomaly detection models, which primarily focus on point, contextual, or global anomalies [15], this problem involves detecting abnormal time series patterns, as defined in [16]. However, there is a significant gap in existing models capable of handling this specific type of anomaly.

In clinical practice, MEP monitoring naturally generates a sequence of discrete evoked responses: following each stimulation, a complete MEP waveform is recorded over a short time window and becomes immediately available to the neurophysiologist. Consequently, the real-time monitoring task consists of assessing each newly acquired MEP waveform individually, as soon as it is recorded, to determine whether it deviates from expected physiological behavior. This approach is limited by the potential error in manual amplitude measurements, the time required to perform them, and fatigue during long procedures [17, 18].

Furthermore, explainability is a critical requirement in this domain, as highlighted in [14]. Deep learning models, while powerful, were unsuitable for our approach due to both the need for interpretability and the challenges associated with training deep networks on small datasets. Instead, we leveraged insights from [12], where ensemble-based methods, particularly Random Forests [19], demonstrated strong performance in classifying MEPs. Building on this evidence, we propose a novel forest-based anomaly detection model tailored to the unique constraints and requirements of intraoperative MEP monitoring.

In this paper, we propose the Canonical Isolation Forest (CIsoF), an extension of Isolation Forest [20] specifically designed for anomaly detection on time series. This model isolates MEPs effectively by leveraging both the temporal structure and statistical properties of these biomedical signals. Our method computes CATCH22 time series features [21], a comprehensive set of statistical and structural descriptors on random intervals of each time series. This effectively captures the characteristics of MEP signals. Additionally, our method is patient-specific, meaning that the model is trained and tested on the same patient, allowing it to account for individual variability in MEP patterns. This patient-specific design ensures robust detection even with the small datasets typical of intraoperative settings. Furthermore, this is the first anomaly detection forest specifically designed for identifying abnormal time series patterns, addressing a gap in existing methods [15]. From an algorithmic perspective, CIsoF continuously assigns a continuous *anomaly score* to each incoming MEP waveform, quantifying its deviation from a patient-specific baseline distribution established at the beginning of the surgical procedure. This formulation naturally leads to an unsupervised anomaly detection setting, which, together with the limited availability of patient data and the clinical requirement for explainable decision mechanisms, motivates the use of forest-based approaches over recent deep learning models in this context. Finally, in addition to standard detection, where the goal is to flag the current MEP as anomalous, we also consider an *early detection* setting. In this scenario, the objective is to provide an anticipatory warning before a clinically confirmed pathological MEP occurs. This is evaluated using the same anomaly scoring mechanism by analyzing whether MEPs immediately preceding a pathological event already exhibit elevated anomaly scores. The rest of the paper is structured as follows. Section 2 discusses related work, including the application of machine learning in intraoperative monitoring and time series anomaly detection, as well as existing forest-based methods for time series. Section 3 presents our proposed method, including the medical problem and its machine-learning formalization, the detection tasks, and details on the Canonical Isolation Tree and Forest. Section 4 provides an extensive experimental evaluation, analyzing different variants of our model, its detection capabilities, and comparisons with alternative methods. Finally, Section 6 concludes the paper, summarizing key findings and potential future directions.

## Related Work

### Machine Learning in Intraoperative Monitoring

The integration of machine learning (ML) into intraoperative monitoring (IOM) has significantly advanced the ability to analyze and interpret neurophysiological data. Traditional methods for monitoring motor evoked potentials (MEPs) rely on empirical thresholds and expert interpretation, which can lead to inconsistencies in decision-making. ML-based approaches offer the potential to enhance the accuracy and robustness of MEP analysis, reducing subjectivity and improving patient safety. A broader review of ML applications in IOM [14] emphasized the role of explainable AI techniques in neurophysiological monitoring. The study underscored the importance of models that not only achieve high accuracy but also provide interpretable decision-making processes, which is essential for clinical adoption. Beyond classification, ML has also been applied to model the effects of anesthetic agents on somatosensory evoked potentials (SSEPs) [22]. By leveraging signal processing and ML techniques, researchers have characterized and predicted SSEP waveform changes under different anesthetic conditions. This suggests that ML models could similarly be employed to predict intraoperative MEP variations and improve anomaly detection. Recent studies have demonstrated the feasibility of ML models for intraoperative MEP classification [12, 23]. For instance, Boaro et al. in [12] developed a machine learning framework that successfully classified MEPs based on the muscles that generated them, surpassing expert-level performance. This work highlights the potential of ML models to assist the surgical team in interpreting MEP alterations more objectively and efficiently while maintaining explainability and reducing complexity. The work of Boaro et al. [12] identified RF as the best-performing model for MEP classification, demonstrating its ability to capture complex patterns within neurophysiological signals.

### Time Series Anomaly Detection and Forest-Based Models

#### Time Series Anomaly Detection

Anomalies in time series data can be broadly categorized into three types: point anomalies, where individual observations deviate significantly from expected values; contextual anomalies, where an observation is anomalous in a specific context but not in others; and collective anomalies, where a group of observations collectively deviates from normal patterns [15]. These anomalies can arise due to equipment failures, environmental changes, or underlying physiological conditions in medical applications. Over the last two decades, the field of time series anomaly detection has been widely explored [24]. This problem can be addressed from five different perspectives: clustering-based, density-estimation-based, distance-based, reconstruction-based, and forecasting-based techniques [15]. Earlier approaches have primarily relied on traditional machine learning [20, 25] and statistical tools [26]. At the same time, more recent methods have explored deep learning (DL) techniques [27, 28], which have been particularly effective in handling high-dimensional time series [16]. However, DL-based methods often require large amounts of training data, making them unsuitable for our problem, where the number of available samples is minimal. In addition to data requirements, deep learning approaches typically offer limited interpretability, which represents a critical limitation in intraoperative neurophysiological monitoring [29–31].

Among the major paradigms, distance-, reconstruction-, and forecasting-based methods explicitly account for the temporal structure of time series data. Distance-based approaches detect anomalies by measuring distances between time series. The most commonly used distance is Dynamic Time Warping (DTW) [32]. Reconstruction-based methods, often leveraging autoencoders or principal component analysis, identify anomalies as instances that cannot be effectively reconstructed from a learned representation. Forecasting-based techniques predict future values and flag anomalies when the actual observations significantly deviate from the expected trajectory [15]. Given the constraints of our datasets, we focus on traditional approaches rather than deep learning solutions due to limited data availability.

Recent research has explored streaming and online anomaly detection frameworks for non-stationary data streams, including adaptive tree-based approaches such as STREAMRHF [33], online detectors designed to handle missing values and concept drift [34], and comprehensive surveys of streaming detection paradigms [35]. These methods are designed for continuously evolving data streams in which the model must update incrementally as new observations arrive and no stable training baseline is available. In contrast, the intraoperative setting studied here is characterized by a well-defined, patient-specific baseline acquired before surgery begins, on which the model is trained once and then kept fixed throughout the procedure. This architectural difference makes streaming update mechanisms unnecessary and potentially harmful. Nevertheless, for settings in which a stable pre-surgical baseline is unavailable, for example, in emergent or unplanned procedures, streaming anomaly detection frameworks represent a meaningful direction for future research.

Building on this background, the following subsection narrows the focus from general time series anomaly detection to the specific methodological foundations most relevant to our approach. Section 2.2.2 reviews forest-based models developed for time series classification, such as Time Series Forest and Canonical Interval Forest, which exploit interval-based feature extraction to capture temporal structure. Although these approaches have shown strong performance in supervised settings, there is still a lack of forest-based methods specifically designed for unsupervised anomaly detection in time series. This limitation motivates the development of CIsoF, which combines the interval-based feature extraction philosophy of canonical forests with an isolation-based anomaly detection strategy, adapted to the patient-specific setting required for intraoperative MEP monitoring.

#### Random Forests for Time Series

In the context of time series classification, forest-based methods have demonstrated strong performance. Time Series Forest (TSF) [36] utilizes intervals of time series to extract features such as mean, standard deviation, and slope, which are then used in decision trees for classification. Canonical Interval Forest (CIF) [37] extends this idea by employing a broader set of summary statistics, improving robustness across different types of time series data. While these models excel in classification tasks, they are not inherently designed for anomaly detection, as they need labeled datasets to be trained.

Despite the effectiveness of existing forest-based methods for classification, a dedicated forest-based anomaly detection model for time series remains absent. In particular, for intraoperative motor evoked potential (MEP) monitoring, anomalies are not merely statistical outliers but deviations from expected physiological signal evolution. Our work fills this gap by proposing a novel forest-based anomaly detection model specifically tailored for time series data, integrating domain-specific adaptations to improve accuracy in this critical application.

## Proposed Methods

In this section, we provide a detailed explanation of our approach to intraoperative MEP anomaly detection. First, we formally define the detection and early detection tasks, establishing the problem formulation both from the medical (see Section 3.1) and computational (see Section 3.2) points of view. Then, in Section 3.3, we introduce our Canonical Isolation Forest (CIsoF), which serves as the foundation for our anomaly detection framework. To avoid ambiguity, we emphasize that the proposed approach is an *unsupervised* task and that the problem belongs to anomaly detection. CIsoF assigns a continuous *anomaly score* to each newly acquired MEP. Throughout the paper, the term “decision” refers to this scoring step.

### Medical Problem

During a major surgical procedure, the patient is under general anesthesia, which eliminates voluntary muscle movement. In those types of surgeries in which monitoring the integrity of the neuromuscular system is needed, such as neurosurgery, vascular surgery or thyroid surgery, the neurophysiologist is responsible for continuously assessing the patient’s motor function by stimulating specific regions of the brain or spinal cord and recording the corresponding muscle responses. These responses, known as MEPs, are collected using electrodes placed on the patient’s skin or in the muscles. At the beginning of the procedure, before any surgical intervention takes place, the neurophysiologist establishes a reference measurement by collecting baseline MEP signals. This baseline represents the expected response of the patient’s motor pathways under normal conditions and serves as a point of comparison for subsequent MEP recordings. It is crucial to ensure that the baseline is stable and reflects the patient’s preoperative neurological status. As the surgery progresses, the neurophysiologist periodically collects new MEPs and compares them to the baseline. The goal of this comparison is to detect any deviations that may indicate potential neurological damage. If the neurophysiologist detects significant changes in the MEP signals, they inform the surgical team. The definition of “significant deviation” mainly refers to signal amplitude reduction. There is no widespread consensus on a specific value, as it varies across studies and depending on the type of surgery, from 20% to complete disappearance. This warning allows the surgical team to take corrective actions, such as temporarily pausing the surgery, modifying patient positioning, adjusting anesthesia parameters, or investigating potential causes of the signal changes. Rapid intervention can help prevent permanent neurological deficits, making intraoperative MEP monitoring a crucial component of patient safety. In the next section (Section 3.2), we will formalize the computational aspects of this detection problem, defining how MEP signals are processed and analyzed algorithmically to achieve robust anomaly detection and early detection. Importantly, intraoperative monitoring produces *discrete* MEP waveforms: after each stimulation, a full MEP trace is acquired within a short time window and becomes immediately available for analysis.

### Machine Learning Formalization

We define a dataset of intraoperative motor evoked potentials (MEPs) as:

1 $$\begin{aligned} \mathcal {D} = \{x_1, x_2, \dots , x_N\} \end{aligned}$$

where *N* is the number of MEPs in $\mathcal {D}$. Each $x_i$ represents a MEP signal:

2 $$\begin{aligned} x_i = \{v_1^i, v_2^i, \dots , v_{l^i}^i\} \end{aligned}$$

where $v_j^i$ denotes the value of the *i*-th signal at time step *j*, and $l^i$ denotes the length of the * i *-th time series.

In our setting, each $x_i$ corresponds to the full waveform of one evoked response (one stimulation), that is, a univariate time series of fixed duration recorded immediately after stimulation.

The dataset $\mathcal {D}$ represents the patient-specific *baseline* and consists of MEP waveforms recorded before a reference time point * λ *, that is, before the surgical procedure begins (baseline acquisition phase). CIsoF is trained once on $\mathcal {D}$ and kept fixed for the remainder of the procedure.

In the next part of this section, we will introduce both the Detection and Early Detection tasks.

After * λ *, a new time series is recorded:

3 $$\begin{aligned} x_{\text {TEST}} = \{v_1, v_2, \dots , v_{l^{\text {TEST}}} \} \end{aligned}$$

where $l^{TEST}$ is the length of the newly observed MEP time series.

The objective is to determine whether $x_{\text {TEST}}$ exhibits anomalous behavior when compared to the learned distribution of normal MEPs in $\mathcal {D}$. Specifically, we seek to develop a model * m * that, given $\mathcal {D}$ and $x_{\text {TEST}}$, outputs a binary decision:

4 $$\begin{aligned} m(\mathcal {D}, x_{\text {TEST}}) = {\left\{ \begin{array}{ll} 1, & \text {if } x_{\text {TEST}} \text { is an anomalous MEP,} \\ 0, & \text {if } x_{\text {TEST}} \text { is a normal MEP.} \end{array}\right. } \end{aligned}$$

An anomalous time series is defined as one that exhibits a significant deviation from the learned distribution of normal MEPs in $\mathcal {D}$, technically as a reduction of at least 20% in signal amplitude relative to the learned baseline. Such deviations are intended to capture potentially pathological intraoperative events that may indicate neurological injury. Our method aims to identify such anomalies early, with the potential to provide real-time alerts to neurophysiologists and surgeons for timely intervention.

**Fig. 1:**
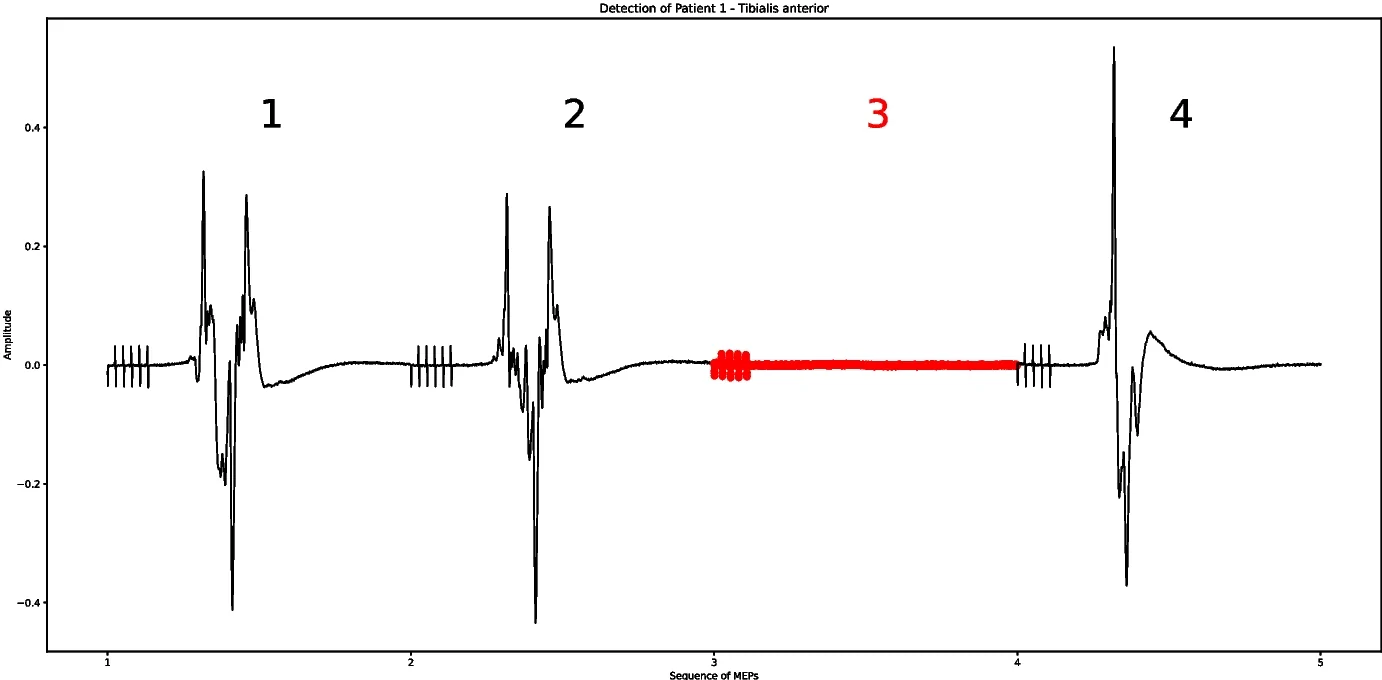
Example of MEP sequences in the testing phase. Each MEP is numbered according to its temporal order. The MEP labeled as #3 (highlighted in red and with a larger line width) is an anomaly. In the detection task, the goal is to determine whether MEP #3 is anomalous

This example in Fig. 1 illustrates a real but straightforward case where the anomaly is visually evident. However, in practical scenarios, distinguishing anomalies is more challenging due to the high variability of MEP signals. Hence, developing robust models capable of accurate detection is crucial for intraoperative neurophysiological monitoring.

CIsoF operates under two distinct tasks: Detection and Early Detection. While the Detection task follows the standard formulation described in Eq. 4, the Early Detection task extends this concept by aiming to predict future anomalies before they manifest. Operationally, the proposed framework assigns a continuous anomaly score to each newly acquired MEP waveform. In the Early Detection task, rather than determining whether the current MEP time series is anomalous, the goal is to predict whether the **next MEP time series** will be anomalous. Formally, given an observed MEP time series $x_t$, the model aims to predict the anomaly status of the subsequent time series $x_{t+1}$, thereby enabling proactive intervention. In this manuscript, we treat early detection as an *anticipatory warning* task evaluated by shifting the ground-truth one step earlier in the MEP sequence (see Section 4.1). Operationally, early detection can be instantiated by thresholding the anomaly score computed on the current MEP $x_t$ and interpreting a high score as a warning that $x_{t+1}$ may become anomalous.

5 $$\begin{aligned} m'(\mathcal {D}, x_t) = {\left\{ \begin{array}{ll} 1, & \text {if } x_{t+1} \text { is expected to be anomalous,} \\ 0, & \text {if } x_{t+1} \text { is expected to be normal.} \end{array}\right. } \end{aligned}$$

The early detection task is particularly crucial in intraoperative monitoring, as it could allow for timely intervention before significant neurological deterioration occurs. By leveraging the temporal structure of MEP sequences, the model can anticipate pathological deviations, reducing the risk of irreversible damage.

### Canonical Isolation Tree and Forest

**Fig. 2:**
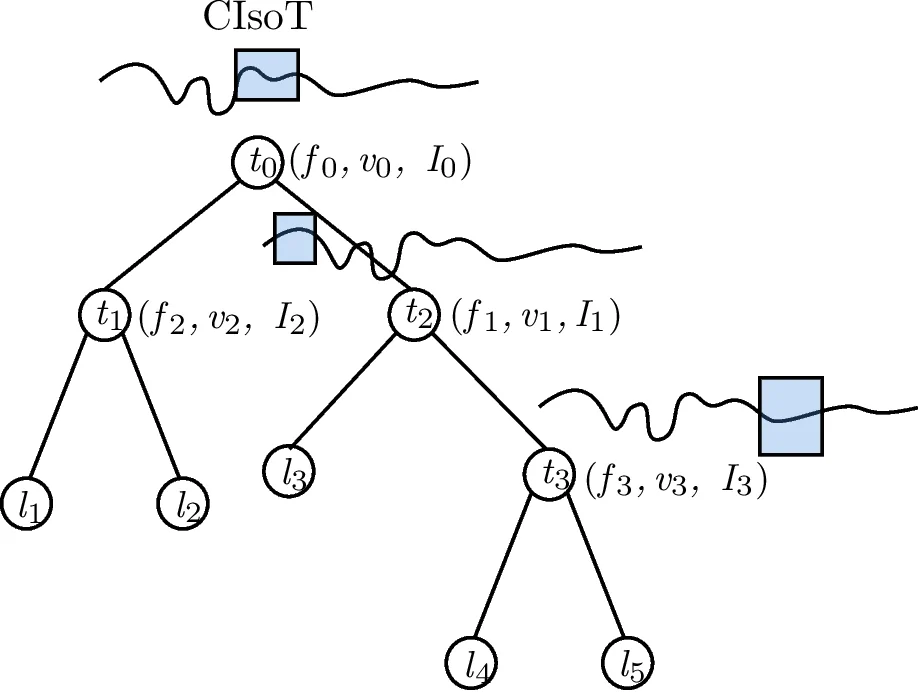
The Canonical Isolation Tree. In each node *n* the decision is taken i) by extracting the interval $I_n$ from the time series (highlighted in light blue); ii) by extracting from it the feature $f_n$; iii) by checking if $x^{f_n}_{I_n} < v_n$, making the time series follow the left branch if true and the right one if false

This section presents the forest-based approach: we first introduce how a tree works and then how to build a forest of this specific tree. The Canonical Isolation Forest (CIsoF) represents an ensemble of Canonical Isolation Trees (CIsoT). A CIsoT is a binary tree structure in which each node has either no children – making it a leaf – or exactly two children – making it an internal node (an illustration is provided in Fig. 2). Internal nodes are associated with tests that determine the routing of a time series: depending on the outcome, the time series is directed to either the left or the right child node. More formally, a test *t* on a time series $x = \{v_1, v_2, \dots , v_{l}\}$ is characterized by a tuple *t=(f,v,I)*, which operates as follows:

- It extracts from the time series *x* the subseries corresponding to the interval defined by *I*. This is the same idea used in Time Series Forest [36] and Canonical Interval Forest [37], where their investigations on classification tasks have shown that using intervals improves predictive performance. Assuming that all time series share the same length, the interval *I* can be specified using absolute positions: $I = [I_{s},I_{e}]$, where $I_s$ and $I_e$ denote the start and end points of the interval, respectively. In cases where the time series may have varying lengths, the interval can instead be defined relative to the series length – for example, by specifying a range from 30% to 35% of the time series. Let us denote as $x_I$ the extracted subseries corresponding to the given interval.
- The feature *f* is extracted from the interval $x_I$, where *f* corresponds to one of the predefined measures (outlined in Table 11). We denote the resulting value as $x^f_I$.
- If $x^f_I < v$, the time series *x* proceeds to the left child; otherwise, it is directed to the right child.

#### Features

The feature *f* is chosen from a set of 25 measures that can be computed from a time series. Specifically, the first 22 features are selected from CATCH22, a well-established collection of 22 descriptive features tailored for time series analysis [21]. For completeness, a summary of the CATCH22 features is provided in the Appendix (see Table 11). This choice is consistent with the approach used in the *Canonical Interval Forest* [37], a recent Random Forest-based method for time series classification. The remaining three features – the mean, the standard deviation, and the slope of the least squares regression line – are adapted from the *Time Series Forest* classifier proposed by Deng et al. [36].

#### Training of a CIsoT

The Canonical Isolation Tree (CIsoT) is constructed using a standard top-down, recursive approach. Beginning with the full training set $\mathcal {D} = \{x_1,..., x_N\}$, consisting of *N* time series, a test *t=(f,v,I)* is selected for the root node. This test partitions $\mathcal {D}$ into two subsets, $\mathcal {D}_L$ and $\mathcal {D}_R$, based on the outcomes of the test for each time series. These subsets are then used to recursively define the tests for the left and right child nodes. The recursion continues until a predefined maximum tree height is reached or until a node contains fewer than a minimum number of samples. Since searching for the "best" question cannot be performed using traditional criteria (such as the Gini criterion in classification trees [38]) due to the absence of labels, we implement two distinct split methods. The first follows the standard random selection approach used in the Isolation Forest [20], where a feature and a split threshold are chosen at random. The second method aims to minimize variance, as proposed in [39]. Specifically, we select a feature and evaluate a limited number of candidate thresholds (as commonly done in the RF paradigm [19]), choosing the one that minimizes the sum of variances in the resulting partitions $\mathcal {D}_L$ and $\mathcal {D}_R$. This variance-minimizing approach divides the data into more homogeneous subsets. More precisely, in our CIsoT, given a set $\mathcal {D}_n$ of time series reaching the node *n*, the test *t=(f,v,I)* of that node is determined in the following way:

- The interval *I* is selected from a group of randomly generated intervals, with a different group created for each tree. Intervals are generated by randomly choosing starting points and lengths^1^.
- The feature *f* is randomly chosen from a randomly selected subset of the 25 predefined features.
- A split threshold *v* in the range $( f_{min}, f_{max} )$ is selected differently based on the split method chosen. Once defined $f_{min} = \min _{j=1\cdots N} (x_{j})_I^f$ and $f_{max} = \max _{j=1,\cdots N} (x_j)_I^f$, we select *v* in two ways^2^:
  - randomly between $$( f_{min}, f_{max} )$$,
  - minimizing the variance of the children by $\arg \min _{v \in (f_{min}, f_{max})} (\sigma ^2_{\mathcal {D}_L} + \sigma ^2_{\mathcal {D}_R})$. The pseudocode is provided in Algorithm 1.

**Algorithm 1:**
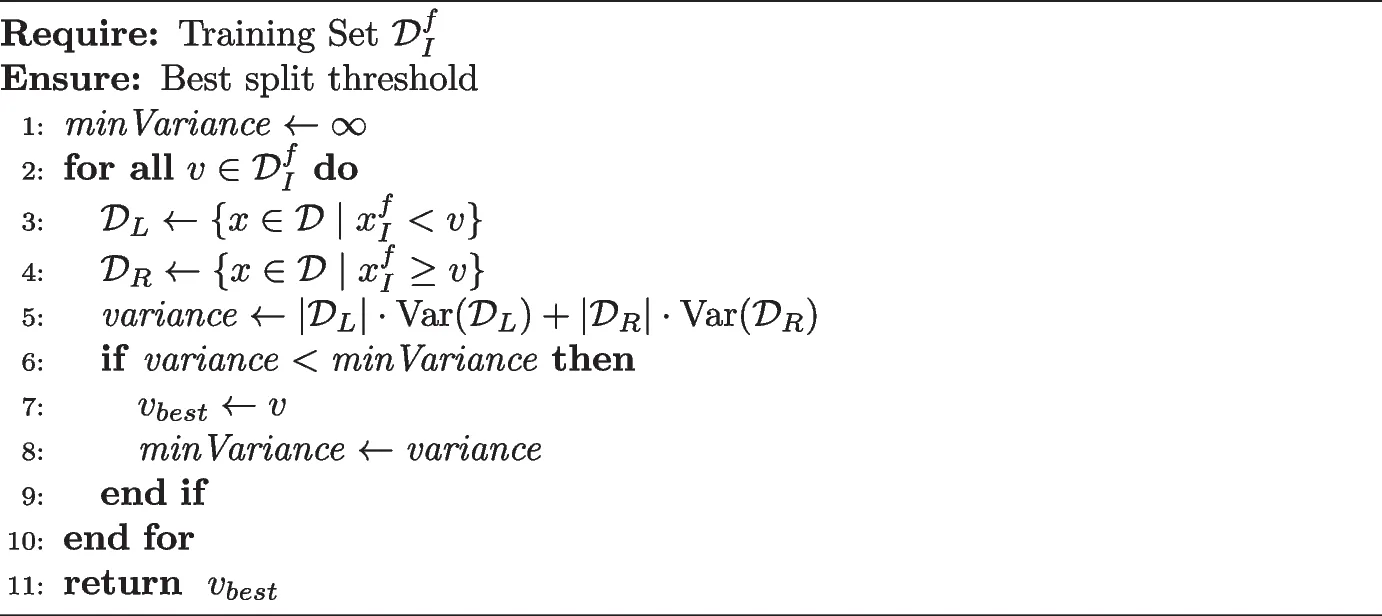
*MinVarianceSplit*: Obtaining best split threshold

The complete training procedure for the CIsoT is provided in Algorithm 2.

**Algorithm 2:**
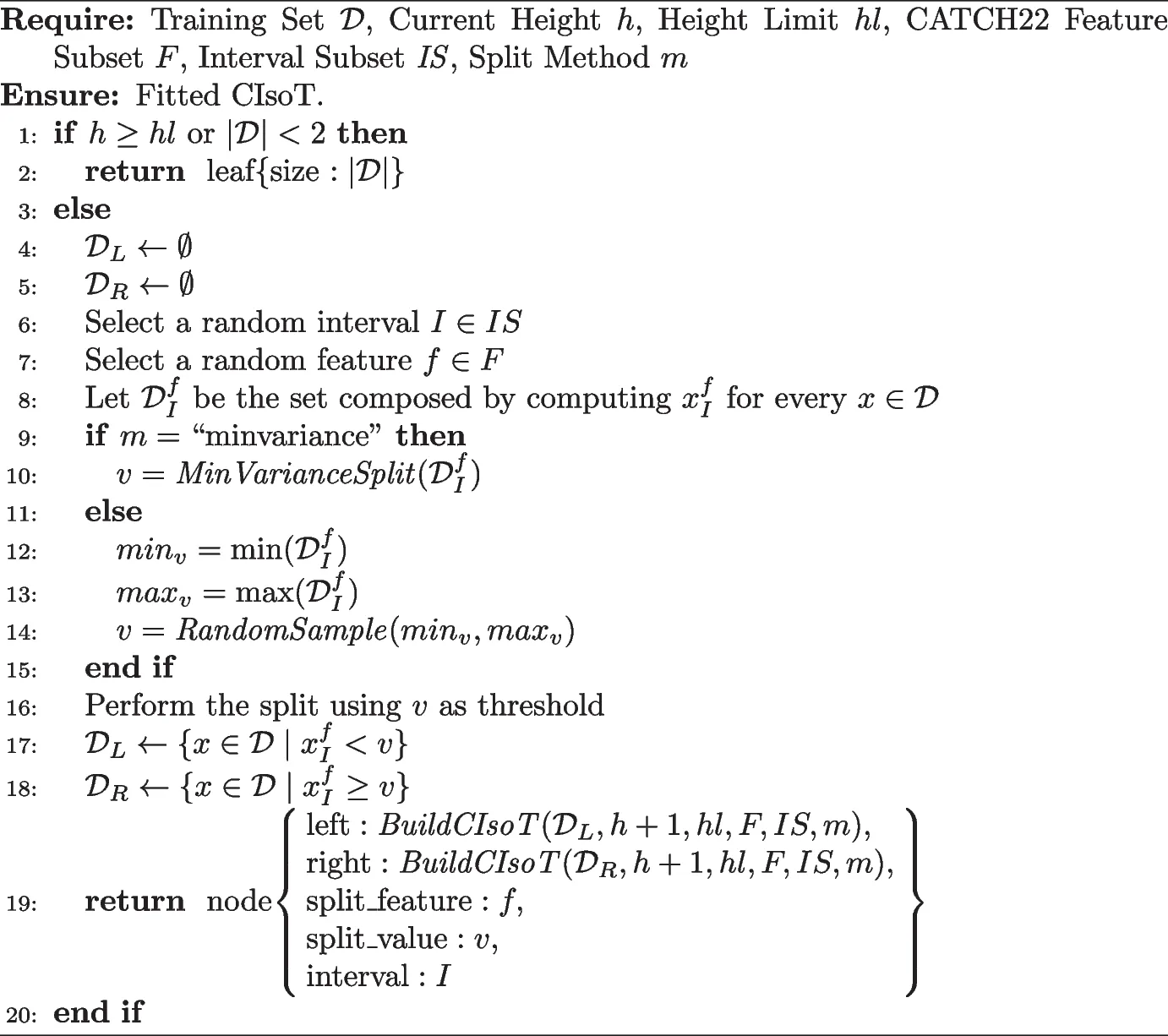
*BuildCIsoT*: Fit a CIsoT

#### Training of a CIsoF

The CIsoF (see Algorithm 3) represents an ensemble of CIsoTs. Following the classical procedure of Isolation Forest, each CIsoT is created using the procedure described previously, starting from a random subsampling of the training set. We set $n_f$, the number of CATCH22 features randomly sampled at each split. This approach aligns with the common practice in the random forest paradigm, where each tree is trained on a random subsample of features to enhance diversity and robustness. Finally, $n_I$ denotes the number of intervals generated for each tree.

**Algorithm 3:**
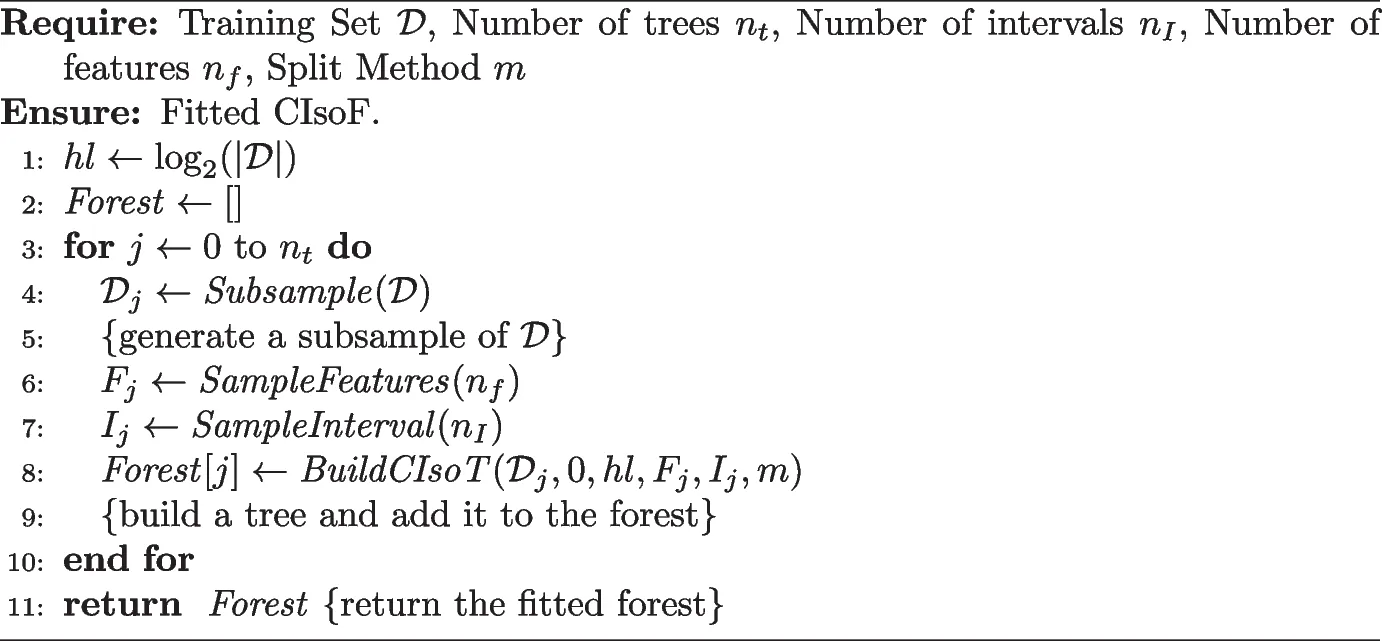
*CIsoFBuild*: Fit a CIsoF

#### Detection of Anomalies

Once the model is trained, we can detect anomalies by thresholding time series according to their anomaly scores, and anomalies are time series that produce a higher score. We define the anomaly score (AS; see Eq. 6) as in the work of Liu et al. [20]. The isolation mechanism exploits the fact that anomalies are easier to isolate than normal instances. Isolation is achieved by recursively partitioning the data using the test splits. Since anomalies are few and different, they tend to be isolated in fewer splits, leading to shorter path lengths in the trees. In contrast, normal instances require more splits due to their higher density and similarity to other points. This fundamental property allows the model to distinguish anomalies based on their expected path length in the constructed trees.

6 $$\begin{aligned} AS(x,n) = 2^{-{\frac{E(h(x))}{c(n)}}} \end{aligned}$$

where *x* is a time series, *n* represents the size of the subsample set used in the training phase, *E*(*h*(*x*)) is the average depth of *x* from a CIsoF, and *c*(*n*) is the average path length of an unsuccessful search in a binary search tree [40].

#### Early Detection of Anomalies

To better understand how the mechanism works for early detection, as detailed in Eq. 5, we retrieve the anomaly score of the MEP that precedes the actual anomaly. By doing so, we aim to distinguish between the anomaly scores of a normal MEP and those of MEPs that occur just before a true anomaly. In this way, we make an early prediction, attempting to anticipate the anomaly before it occurs, as detailed in Section 4.1. Early detection performance depends on the degradation modality of the signal. With gradual signal degradation, the MEP recorded just before the confirmed anomaly is already different from baseline. This means that an anomalous signal can be detected earlier, before it is fully confirmed. In contrast, when the pathological event occurs abruptly, no detectable precursor is present, making anticipatory detection inherently more difficult. The early detection score should therefore be interpreted as a soft warning signal requiring clinical confirmation, not an autonomous alarm.

The proposed approach also differs from forecasting-based methods, which estimate future anomaly scores by fitting a model to the history of past scores. In the intraoperative setting, the pre-anomaly baseline is too short and non-stationary for reliable forecasting. Instead, an elevated anomaly score on $x_t$ directly reflects how much the current physiological state deviates from the patient baseline, providing a natural and interpretable precursor signal for $x_{t+1}$ without requiring a separate predictive model.

## Experimental Details

### Dataset Creation

The dataset used in this study consists of data collected from four patients undergoing neurosurgical procedures for intramedullary spinal cord tumor removal. The protocol used to elicit MEPs was transcranial electrical stimulation (TES). MEPs were evoked by applying transcranial anodal electrical stimulation with corkscrew electrodes placed on the scalp at C1 and C2 according to the 10–20 International System. The high-frequency short-train technique was used, applying a train of five pulses with an interstimulus interval of 4 ms. Intensity ranged between 50 and 150 mA. MEPs were recorded from the same target muscles in all patients, with pairs of subdermal needle electrodes placed in the muscle belly of the abductor pollicis brevis (APB) for upper limbs and the tibialis anterior (TA) and abductor hallucis (AH) for lower limbs. All procedures were performed in compliance with institutional guidelines and were approved by the appropriate institutional committee. The study obtained IRB approval with protocol ID: CRMS 23038 on June 05, 2024. These muscles were chosen because they were the only ones consistently monitored across all patients, ensuring uniformity in the dataset.

For each patient–muscle pair, we consider the chronological sequence of MEPs acquired during the procedure. Each MEP waveform corresponds to one stimulation and is treated as one univariate time series instance (see Section 3.2). The goal is to train a patient-specific baseline model using only the initial, stable portion of the sequence and then evaluate detection performance on the subsequent MEPs acquired during surgery.

The methodology for splitting the signals was designed to closely simulate real-world conditions in the operating room. The first anomaly within the sequence of MEP signals recorded during the procedure was identified. Using this anomaly as a reference point, the dataset was divided into two parts: the MEP signals preceding the first anomaly constituted the training dataset, while those following it, including the anomaly itself, formed the testing dataset. This approach preserves the natural chronological order of events, ensuring that the training and testing datasets reflect real-time clinical scenarios.

#### Early detection dataset

To evaluate whether CIsoF provides a warning signal in advance of a clinically confirmed anomalous MEP, we construct an “early detection” version of each dataset by shifting the anomaly label one step earlier in the sequence. Concretely, for each anomaly occurring at time index *t*, we label the preceding MEP at index $t{-}1$ as positive (early warning), and exclude the MEP at *t* from the evaluation set for that specific event. In practice, this is implemented by removing the first MEP in the detection test set and replacing it with the last baseline MEP, and then repeating the procedure sequentially for each subsequent anomaly.

In Tables 1 and 2, a summary of the datasets is reported for both the Detection and Early Detection tasks, respectively. For each cell, the first number is the number of samples in the training set, the second number is the number of samples in the test set, and the third number is the proportion of anomalies in the test set.

**Table 1:** Detection Cardinality (Train-Test samples and percentage of anomalies in Test set)

	AH	APB	TA
1	130-366(0.16)	25-94(0.06)	130-111(0.12)
2	6-249(0.17)	6-332(0.17)	33-616(0.01)
3	7-25(0.36)	7-151(0.01)	19-77(0.12)
4	11-868(0.13)	41-1571(0.04)	7-153(0.29)

**Table 2:** Early Detection Cardinality (Train-Test samples and percentage of anomalies in Test set)

	AH	APB	TA
1	129-308(0.19)	24-89(0.07)	129-99(0.13)
2	5-207(0.21)	5-275(0.21)	32-609(0.01)
3	6-17(0.53)	6-150(0.01)	18-69(0.13)
4	10-757(0.15)	40-1505(0.04)	6-109(0.41)

### CIsoF in Detail

In this section, we outline the experimental setup used to evaluate our proposed method, detailing the different configurations, split methods, evaluation metric, and repetition strategy employed. We designed three configurations for CIsoF to evaluate the trade-off between computational efficiency and predictive performance. We used the **H**eavy, **L**ight, and **U**ltra**L**ight configurations:

- In the **H**eavy configuration, we set the number of trees ($n_t$) to 500, the number of features ($n_f$) to 4, and the number of intervals ($n_I$) to $\sqrt{N}$, where *N* is the sequence length.
- In the **L**ight configuration, $n_t=200$, $n_f=4$, and $n_I = \log _2{N}$.
- In the **U**ltra**L**ight configuration, $n_t=100$, $n_f=4$, and $n_I = \log _{10}{N}$.

We evaluated two different split methods (both described in Section 3.3):

1. ***Random***: standard method in Isolation Forest [20].
2. ***MinVariance***: unsupervised split technique that can be optimized.

To evaluate performance, we used the Receiver Operating Characteristic area under the curve (ROC-AUC), a widely used metric for anomaly detection problems [20, 41]. For all statistical tests, we set *α = 0.05*. Finally, to assess the impact of randomization on model performance, we conducted all experiments ten times for each dataset, configuration, and split method, and report the average over these repetitions. This repetition allows us to measure how inherent randomization affects the performance of our proposed method.

## Results and Discussion

### Analysis of CIsoF Variants

As an initial analysis, we compared the different configurations of CIsoF to evaluate their performance and computational efficiency. Table 3 presents a comparison of the three configurations in terms of their performance in detection and early detection tasks; results are averages of ROC-AUC across patients and muscles. The **H**eavy configuration achieves the highest performance across both tasks, while the **U**ltra**L**ight and **L**ight configurations exhibit similar scores. The **L**ight configuration provides a balance between computational efficiency and predictive performance, improving upon the **U**ltra**L**ight configuration on the detection task while maintaining a reduced computational burden compared to the **H**eavy configuration.

**Table 3:** Mean ROC-AUC comparison of configurations (UL, L, H) for detection and early detection

	UL	L	H
Detection	0.6681	0.6781	0.7824
Early Detection	0.5403	0.5257	0.6077

**Fig. 3:**
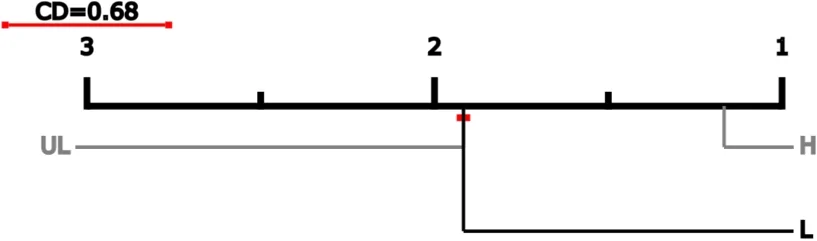
Critical difference diagram comparing the ROC-AUC of the three configurations: **UL**, **L**, and **H** over all datasets

To assess the statistical significance of our comparisons, we conducted a Friedman test, followed by a post-hoc pairwise analysis using Nemenyi’s test for multiple comparisons of mean rank sums [42]. The Friedman test determines whether there are significant overall differences in the rankings of the evaluated methods across different datasets. If a significant difference is found, Nemenyi’s test is applied to examine pairwise differences in mean ranks. The results are then visualized using a critical difference diagram (see Fig. 3), where the mean ranks of the methods across datasets are displayed. The red lines connect rankings that do not present statistically significant differences.

Figure 3 shows the critical difference diagram, where configurations are ranked based on their average performance across datasets. The numbers 1–3 indicate the average ranks, increasing from best to worst (left to right). Two configurations must differ by at least 0.68 (critical difference) to be considered statistically different. The **L**ight and **U**ltra**L**ight configurations, which yield the lowest performance, do not differ in a statistically significant manner. In contrast, the **H**eavy configuration is statistically distinct from both. Overall, the **H**eavy configuration represents a suitable compromise when performance is the primary concern.

Given this statistically superior performance, it is important to assess whether the **Heavy** configuration remains compatible with real-time intraoperative deployment. Inference is highly efficient and performed exclusively at test time, while model training is carried out offline. On a consumer-grade laptop CPU (Intel^®^ Core^™^ i9-10980HK), CIsoF requires approximately 2–3 ms to score a single MEP, enabling the processing of several hundred MEPs per second. In practical terms, batch inference over 10–50 MEPs achieves throughputs between approximately 300 and 430 MEPs/s, which is orders of magnitude faster than the rate at which MEPs are acquired during surgery. These results demonstrate that the **Heavy** configuration can be safely employed in real-time intraoperative monitoring scenarios.

In the next part, we present the performance of CIsoF models for both detection and early detection tasks, taking the best among the configurations. Two tables summarize the results: Table 4 for detection, and Table 5 for early detection; each table includes both split methods described above. We denote the Random and MinVariance variants as CIsoF_R_ and CIsoF_MV_, respectively. Each table evaluates the models over four patients, denoted as rows 1–4, and the three muscle groups described above. Overall, the proposed model performs well, achieving an ROC-AUC greater than 0.80 in 12 out of 24 datasets in the Detection task, as shown in Table 4. Additionally, the MinVariance variant demonstrates strong performance, achieving ROC-AUC scores above 0.80 across 8 datasets. In the next section, we provide a detailed analysis of these results to further examine the model’s behavior across different scenarios. Table 4 (left) reports the Random split variant, CIsoF_R_. The highest scores, on average, are observed for AH, with values ranging from 0.7028 to 0.9652. TA shows high average performance but variable performance across patients. However, APB shows lower scores, particularly in patients 2 and 4, where it falls around 0.50. Table 4 (right) reports the MinVariance split variant, CIsoF_MV_, aimed at improving the quality of child nodes. This modification leads to substantial performance gains across most metrics, particularly for APB. For instance, APB improves from 0.3466 for patient 3 to 0.8634. For TA, performance improves in two of four patients, with a mean ROC-AUC of approximately 0.88. These results suggest that minimizing the variance in splits better captures the structure of the underlying data, leading to improved detection performance. Table 5 evaluates the models’ ability to perform early detection. The left subtable reports the Random split variant. While AH and TA maintain reasonable scores (e.g., 0.8262 and 0.7188 for patient 1), the performance for APB is relatively low, particularly in patients 2 and 4, with scores around 0.55.

**Table 4:** CIsoF ROC-AUC for detection under the two split methods

	AH	APB	TA
Random split			
1	0.9652	0.7078	0.9827
2	0.9619	0.4922	0.5389
3	0.7028	0.3466	0.9757
4	0.7539	0.5360	0.7736
MinVariance split			
1	0.9707	0.8985	0.9817
2	0.9382	0.5852	0.8909
3	0.7931	0.8634	0.9871
4	0.9596	0.6102	0.6411

The right subtable reports the MinVariance split criterion for early detection. Compared to the Random split, APB improves in two of four patients (e.g., 0.6402 to 0.9672 in patient 3), while TA improves in three of four patients.

**Table 5:** CIsoF ROC-AUC for early detection under the two split methods

	AH	APB	TA
Random split			
1	0.8262	0.6036	0.7188
2	0.6891	0.5616	0.5516
3	0.6292	0.6402	0.5426
4	0.5855	0.5431	0.5675
MinVariance split			
1	0.8136	0.6675	0.7262
2	0.6441	0.5282	0.6058
3	0.6611	0.9672	0.6919
4	0.6620	0.4956	0.5204

Compared to the detection task, where our model achieved strong performance with high ROC-AUC scores, the early detection task proved to be more challenging, as expected. The Random split variant exceeds a ROC-AUC of 0.60 in 6 out of 12 datasets, while the MinVariance variant does so in 9 out of 12 cases. These results are less consistent than in the detection setting but still indicate promising predictive capability in this more difficult scenario, where identifying anomalies at an earlier stage is inherently challenging.

We compared the two different split methods of CIsoF, namely ***Random*** and ***MinVariance***. Specifically, we averaged the results across the three previously described configurations and all datasets. These results are presented in Table 6. To determine whether the observed differences were statistically significant, we applied the Wilcoxon signed-rank test [43], a non-parametric test that evaluates whether two paired samples drawn from related distributions differ significantly. The test results indicate a significant difference between the ***MinVariance*** and ***Random*** splitting strategies. Moreover, ***MinVariance*** consistently outperformed ***Random***, confirming its statistical superiority.

**Table 6:** Mean ROC-AUC comparison of split methods (Random and MinVariance) for detection and early detection

	Random	MinVariance
Detection	0.6645	0.7546
Early Detection	0.5485	0.5673

### Comparison with Literature Alternatives

In this section, we present the results of our experiments evaluating different approaches [41] for abnormal time series detection. Since there is no standard method for solving this problem, we compare our proposed approach against alternative strategies. We found the problem described in the literature in [16], but, as already explained in the introduction, deep learning techniques are unsuitable for our case. Thus, we explored two approaches to tackling this problem. The first involves transforming time series into feature vectors using statistical and temporal descriptors, in our case CATCH22 [21], and then applying standard anomaly detection models. The second approach relies on computing distances between time series — in our case, using constrained Dynamic Time Warping (cDTW) with a constraint value of 5% of the time series length — and subsequently employing a distance-based anomaly detection model. The selected models cover a diverse range of anomaly detection methodologies. One-Class Support Vector Machine (OC-SVM) [44] follows a boundary-based approach, learning a hyperplane that encapsulates normal data distribution in a high-dimensional space. Subspace Outlier Detection (SOD) [45] is designed for high-dimensional datasets, detecting anomalies within relevant subspaces. LOF-cDTW [46, 47] extends the classic Local Outlier Factor (LOF) by incorporating cDTW as a dissimilarity measure for time series data. Histogram-Based Outlier Score (HBOS) [48] is a non-parametric method that models feature distributions using histograms and assigns anomaly scores based on deviations from expected densities. Empirical Cumulative Distribution Outlier Detection (ECOD) [49] is a fully unsupervised approach that estimates the empirical cumulative distribution function and detects anomalies based on extreme deviations. Finally, Minimum Covariance Determinant (MCD) [50] is a robust statistical technique that identifies anomalies by computing the most representative subset of the data and detecting points that significantly deviate from it. We utilized the implementations available in the PyOD Python library (https://pyod.readthedocs.io/en/latest/index.html), which provides a comprehensive suite of anomaly detection tools.

Table 7 presents the ROC-AUC comparison between our proposed model and the other anomaly detection methods on the Detection Task. Our approach outperforms all competitors in 10 out of 12 cases, demonstrating its superior performance across most scenarios. Among the alternative models, ECOD achieves the second-best results overall, performing best in 2 cases.

**Table 7:** ROC-AUC comparison for detection across models

		IForest	OCSVM	SOD	LOF-cDTW	HBOS	ECOD	MCD	CIsoF$_{\text {R}}$	CIsoF$_{\text {MV}}$
1	AH	0.8965	0.8908	0.2195	0.5062	0.8270	0.8551	0.7378	0.9652	0.9707
	APB	0.2767	0.4110	0.5663	0.3617	0.2841	0.6951	0.5718	0.7078	0.8985
	TA	0.9169	0.7245	0.6468	0.6601	0.8132	0.9474	0.9195	0.9827	0.9817
2	AH	0.7832	0.6885	0.4081	0.8395	0.4525	0.7230	0.6841	0.9619	0.9382
	APB	0.3435	0.5000	0.6330	0.5631	0.2263	0.8083	0.5654	0.4922	0.5852
	TA	0.6135	0.3639	0.3520	0.1053	0.8131	0.8106	0.4106	0.5389	0.8909
3	AH	0.4635	0.5000	0.4583	0.2292	0.7014	0.5139	0.5736	0.7028	0.7931
	APB	0.1374	0.5034	0.2148	0.0235	0.2114	0.8322	0.3440	0.3466	0.8634
	TA	0.6964	0.5441	0.6340	0.2304	0.4085	0.8431	0.4606	0.9757	0.9871
4	AH	0.4198	0.5354	0.5736	0.4115	0.3308	0.5514	0.6993	0.7539	0.9596
	APB	0.6076	0.6160	0.4414	0.5524	0.7327	0.7608	0.6969	0.5360	0.6102
	TA	0.6170	0.5048	0.4866	0.5321	0.6121	0.5918	0.5607	0.7736	0.6411
Mean		0.5643	0.5652	0.4695	0.4179	0.5344	0.7444	0.6020	0.7281	0.8433

Table 8 presents the ROC-AUC comparison for the Early Detection Task, which is the most critical evaluation in our study. CIsoF achieves the highest performance in 8 out of 12 cases. In contrast, ECOD wins 3 times, while MCD achieves the highest score in a single case. This further reinforces the advantage of our approach over traditional anomaly detection methods. On average, CIsoF_MV_achieves a mean ROC-AUC of 0.6653, which is approximately 6% higher than ECOD’s mean score of 0.6081, further highlighting its superior effectiveness in early anomaly detection.

**Table 8:** ROC-AUC comparison for early detection across models

		IForest	OCSVM	SOD	LOF-cDTW	HBOS	ECOD	MCD	CIsoF$_{\text {R}}$	CIsoF$_{\text {MV}}$
1	AH	0.5900	0.6778	0.6072	0.6784	0.5664	0.6315	0.6571	0.8262	0.8136
	APB	0.5534	0.5843	0.6747	0.4297	0.5341	0.6847	0.6671	0.6036	0.6675
	TA	0.5794	0.4267	0.5653	0.5818	0.6011	0.6950	0.5699	0.7188	0.7262
2	AH	0.5982	0.5693	0.5067	0.4558	0.5125	0.6792	0.5732	0.6891	0.6441
	APB	0.3537	0.5000	0.6437	0.3983	0.4769	0.6920	0.6027	0.5616	0.5282
	TA	0.4342	0.3696	0.7169	0.4056	0.6624	0.6741	0.3044	0.5516	0.6058
3	AH	0.4542	0.5000	0.7778	0.6250	0.6597	0.5833	0.5319	0.6292	0.6611
	APB	0.0639	0.5034	0.4932	0.0456	0.0270	0.5541	0.2514	0.6402	0.9672
	TA	0.5561	0.5500	0.5352	0.4639	0.4444	0.5148	0.6626	0.5426	0.6919
4	AH	0.3959	0.4978	0.4482	0.2720	0.4208	0.4737	0.4980	0.5855	0.6620
	APB	0.5086	0.5281	0.4987	0.5363	0.5009	0.5764	0.5819	0.5431	0.4956
	TA	0.5137	0.5042	0.5396	0.4997	0.5528	0.5382	0.4963	0.5675	0.5204
Mean		0.4668	0.5176	0.5839	0.4493	0.4966	0.6081	0.5331	0.6216	0.6653

To statistically assess the performance of CIsoF, we selected its best variant (CIsoF_MV_) and compared it against the best competitor ECOD. We then conducted a Wilcoxon signed-rank test to evaluate whether CIsoF_MV_significantly outperforms the alternative. The test results confirmed that CIsoF_MV_passed the significance threshold, demonstrating that it is statistically superior to the best competing model.

### External Cohort Validation

To further validate the generalization capability of the proposed CIsoF method, we conducted a comprehensive evaluation on an independent external cohort of patients. The external validation cohort comprised MEP recordings from 13 patients who underwent neurosurgical procedures to the spinal cord. Importantly, these data were collected prospectively without prior knowledge of our study’s specific methodological assumptions. The recordings were obtained from two consistently monitored muscles across all patients: the abductor pollicis brevis (APB) for upper limb monitoring and tibialis anterior (TA) for lower limb assessment. For completeness, the cardinality of the dataset used for both the detection and early detection (see Tables 12 and 13, respectively) tasks in this external cohort is reported in Appendix B.

#### Performance Analysis Across External Cohort

Table 9 presents the comparison of CIsoF configurations (UltraLight, Light, and Heavy) on the external cohort, stratified by task type. Results demonstrate that the Heavy configuration continues to achieve superior performance in the external setting, with a detection ROC-AUC of 0.7988 and an early detection score of 0.5172. This consistency with the primary analysis validates the appropriateness of the Heavy configuration across diverse clinical datasets.

**Table 9:** Mean ROC-AUC comparison of configurations (UL, L, H) for detection and early detection

	UL	L	H
Detection	0.7022	0.7541	0.7988
Early Detection	0.4971	0.4815	0.5172

The detection task achieved a mean ROC-AUC of 0.7988 for the Heavy configuration, representing strong anomaly detection capability in previously unseen patient populations. The early detection task, while more challenging as anticipated, achieved a mean ROC-AUC of 0.5172, demonstrating that the temporal predictive capability translates reasonably well to external data.

The comparison between Random and MinVariance split methods in the external cohort (Table 10) replicates the findings from the primary analysis. The MinVariance approach substantially outperformed the Random method for both detection (0.7923 vs. 0.7111) and early detection (0.5104 vs. 0.4868), yielding performance improvements. These results strongly validate that variance-minimizing split criteria provide superior partitioning in unsupervised anomaly detection, when applied to previously unobserved clinical data.

**Table 10:** Mean ROC-AUC comparison of split methods (Random and MinVariance) for detection and early detection

	Random	MinVariance
Detection	0.7111	0.7923
Early Detection	0.4868	0.5104

**Fig. 4:**
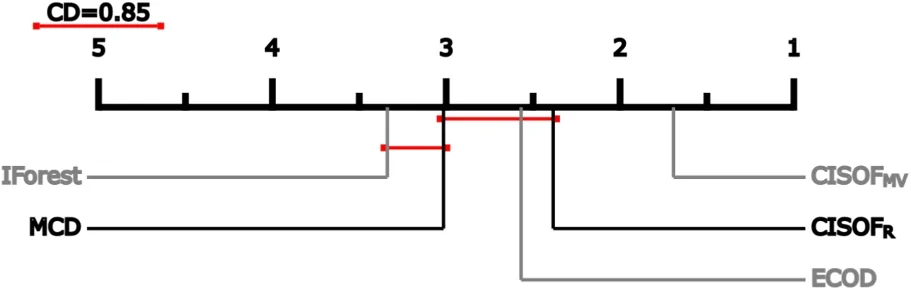
Critical difference diagram comparing the top five anomaly detection models based on mean ROC-AUC across all external cohort datasets. Models are ranked from left (worst) to right (best) by average rank. CIsoF_MV_ achieves the best average rank and is statistically superior to all competing methods

Figure 4 presents the critical difference diagram derived from Friedman test analysis and Nemenyi post-hoc pairwise comparisons, ranking the five best-performing models according to their mean ROC-AUC across all external cohort datasets.

First, CIsoF achieves the best average rank among all evaluated methods, demonstrating superior overall performance in the external validation setting. Specifically, the variance-minimizing variant (CIsoF_MV_) consistently outperforms competing approaches, including OC-SVM, SOD, LOF-cDTW, HBOS, ECOD, and MCD. The statistical analysis confirms that CIsoF is statistically different from the competing methodologies at the *α = 0.05* level, indicating that the observed performance advantage is not attributable to random variation.

#### Detailed Performance Across Patient Populations

A granular analysis of the top five models’ performance across individual patients and muscles is provided in Table 14 (Appendix Section C). CIsoF_MV_ achieves ROC-AUC exceeding 0.90 in 14 out of 26 detection datasets, demonstrating reliable high-performance anomaly detection in the majority of external cohort patients. Even in more challenging datasets, CIsoF_MV_ generally maintains competitive performance for detection tasks, falling below 0.60 in only one case.

Early detection performance, while more variable, remains above chance levels (0.50) in most datasets, with CIsoF_MV_ achieving a mean early detection ROC-AUC of 0.57 across the external cohort. The greater variability in early detection reflects the inherent difficulty of predicting future anomalies from current observations.

### Limitations and Future Directions

From a clinical perspective, false positives and false negatives carry asymmetric costs during intraoperative monitoring. A missed detection (false negative) may delay intervention during neurological deterioration, potentially leading to irreversible motor deficits. Conversely, an excessive rate of false alarms (false positives) may reduce the practical usability of the system, increase the cognitive burden on the surgical team, and ultimately undermine trust in the warning signal.

The proposed framework addresses this asymmetry through its continuous anomaly score output. Rather than imposing a fixed binary threshold, the system allows the operating threshold to be selected by the neurophysiologist at the beginning of the procedure, according to the risk profile of the specific surgical context. In high-risk procedures, a lower threshold (higher sensitivity) can be adopted to minimize missed detections at the cost of a higher false alarm rate; in lower-risk settings, a stricter threshold reduces false alarms. The ROC-AUC metric used throughout this work characterizes this trade-off across all possible operating points precisely, making it an appropriate evaluation criterion for a system intended to support, rather than replace, clinical judgment. In all cases, the anomaly score is presented to the neurophysiologist as an assistive signal, and all clinical decisions remain the sole responsibility of the surgical team.

Several limitations should nonetheless be acknowledged. First, the study is based on a limited number of patients, reflecting the intrinsic difficulty of collecting intraoperative MEP data. While our results are encouraging, larger and more diverse cohorts are required to fully assess robustness across surgical types, anatomical targets, and anesthetic protocols. Second, early detection is evaluated retrospectively through an anticipatory labeling protocol; prospective validation is therefore necessary to confirm the utility of early warning signals in real-time surgical decision-making. Third, before clinical implementation can be considered viable, the physiological meaning of the mathematical features involved should be further explored and defined.

Future work will focus on prospective clinical validation, integration with multimodal intraoperative monitoring signals, adaptive threshold selection strategies, and robustness to non-pathological physiological variations. Addressing these directions is essential to bridge the gap between methodological development and the safe, effective deployment of the system in clinical practice.

## Conclusions

This study presents a novel approach to the detection and early detection of pathological intraoperative motor evoked potentials by introducing the Canonical Isolation Forest (CIsoF), an extension of Isolation Forest specifically tailored to time series biomedical signals. Our results demonstrate that the proposed method effectively identifies deviations from patient-specific baseline MEP patterns and, in cases characterized by gradual neurological compromise, provides early warning signals prior to clinically confirmed pathological events.

A key strength of the proposed framework is its compatibility with real intraoperative constraints. CIsoF operates in a patient-specific, unsupervised manner, requires only baseline data acquired at the beginning of the procedure, and produces interpretable anomaly scores without relying on real-time labeling, online retraining, or automated decision-making. The proposed framework represents a promising step toward computational support tools for intraoperative neurophysiological monitoring, while additional prospective and clinical validation studies remain necessary before practical deployment can be considered.

## Data Availability

No datasets were generated or analysed during the current study.

## Appendix A CATCH22 Features

**Table 11:** Simplified descriptions of the CATCH22 features grouped by category, based on [21] and following the table format proposed in [51]

Category	Description
Distribution	*∙ * Mode of z-scored distribution (5-bin histogram)
	*∙ * Mode of z-scored distribution (10-bin histogram)
Simple temporal statistics	*∙ * Longest period of consecutive values above the mean
	*∙ * Time intervals between successive extreme events above the mean
	*∙ * Time intervals between successive extreme events below the mean
Linear autocorrelation	*∙ * First 1/*e* crossing of autocorrelation function
	*∙ * First minimum of autocorrelation function
	*∙ * Total power in lowest fifth of frequencies in the Fourier power spectrum
	*∙ * Centroid of the Fourier power spectrum
	*∙ * Mean error from a rolling 3-sample mean forecasting
Nonlinear autocorrelation	*∙ * Time-reversibility statistic, $\langle (x_{t+1}-x_t)^3\rangle _t$
	*∙ * Automutual information, *m=2*, *τ =5*
	*∙ * First minimum of the automutual information function
Successive differences	*∙ * Proportion of successive differences exceeding 0.04*σ * [52]
	*∙ * Longest run of successive incremental decreases
	*∙ * Shannon entropy of two successive letters in equiprobable 3-letter symbolization
	*∙ * Change in correlation length after iterative differencing
	*∙ * Exponential fit to successive distances in 2-d embedding space
Fluctuation Analysis	*∙ * Proportion of slower timescale fluctuations that scales with DFA (Detrended Fluctuational Analysis) (50% sampling)
	*∙ * Proportion of slower timescale fluctuations that scales with linearly rescaled range fits
Others	*∙ * Trace of covariance of transition matrix between symbols in 3-letter alphabet
	*∙ * Periodicity measure, as defined in [53]

## Appendix B External Cohort Cardinality

**Table 12:** Detection Cardinality (Train-Test samples and percentage of anomalies in Test set)

	APB	TA
1	39-976(0.03)	44-711(0.07)
2	21-457(0.14)	21-607(0.00)
3	6-274(0.32)	335-840(0.05)
4	25-1817(0.01)	7-586(0.08)
5	8-56(0.29)	7-41(0.37)
6	7-189(0.14)	7-526(0.02)
7	12-952(0.17)	457-1484(0.02)
8	122-58(0.03)	13-9(0.56)
9	97-188(0.11)	9-198(0.20)
10	34-39(0.15)	9-26(0.38)
11	7-173(0.12)	18-47(0.30)
12	14-75(0.09)	7-48(0.33)
13	15-162(0.18)	15-230(0.15)

**Table 13:** Early Detection Cardinality (Train-Test samples and percentage of anomalies in Test set)

	APB	TA
1	38-946(0.03)	43-660(0.08)
2	20-396(0.16)	20-606(0.00)
3	5-188(0.46)	334-796(0.06)
4	24-1807(0.01)	6-540(0.09)
5	7-41(0.39)	6-27(0.56)
6	6-163(0.17)	6-518(0.02)
7	11-789(0.21)	456-1455(0.02)
8	121-57(0.04)	12-5(1.00)
9	96-168(0.12)	8-159(0.25)
10	33-34(0.18)	8-17(0.59)
11	6-153(0.14)	17-34(0.41)
12	13-69(0.10)	6-33(0.48)
13	14-134(0.22)	14-197(0.17)

## Appendix C External Cohort Complete Results

Table 14 reports the detailed performance results for each dataset, listing the five best-performing models. These results further confirm the robustness of CIsoF, which consistently achieves strong detection and early detection performance despite the increased variability introduced by the new patient cohort.

**Table 14:** Top 5 anomaly detection models

			MCD	SOD	ECOD	CIsoF_R_	CIsoF_MV_
1	APB	D	0.2767	0.5718	0.6951	0.7208	0.9011
		ED	0.5534	0.6671	0.6847	0.6757	0.6295
	TA	D	0.9121	0.9050	0.9274	0.9763	0.9814
		ED	0.6468	0.6208	0.7203	0.7521	0.7956
11	APB	D	0.4395	0.6621	0.5599	0.7473	0.7670
		ED	0.5161	0.5533	0.5369	0.6279	0.6860
	TA	D	0.9638	0.9685	0.9042	0.9732	0.9835
		ED	0.7964	0.8292	0.8033	0.8217	0.8137
13	APB	D	0.8019	0.7760	0.9307	0.8567	0.9380
		ED	0.5278	0.5080	0.5570	0.5285	0.4403
	TA	D	0.4090	0.3689	0.6683	0.5563	0.7819
		ED	0.4570	0.4912	0.5923	0.5783	0.6898
14	APB	D	0.6425	0.7023	0.5234	0.5359	0.6225
		ED	0.4490	0.4928	0.5150	0.4530	0.5893
	TA	D	0.6758	0.5615	0.5641	0.5872	0.6269
		ED	0.5706	0.6200	0.6722	0.4506	0.6300
17	APB	D	0.7623	0.6123	0.6808	0.7609	0.9010
		ED	0.4153	0.4350	0.4856	0.5306	0.5899
	TA	D	0.3753	0.3955	0.7182	0.5034	0.7299
		ED	0.2959	0.3239	0.4894	0.4978	0.4297
18	APB	D	0.7771	0.6811	0.5891	0.8163	0.9014
		ED	0.6436	0.6300	0.5195	0.6975	0.8162
	TA	D	0.7728	0.7224	0.9285	0.9874	0.9942
		ED	0.4683	0.6203	0.5804	0.6365	0.6375
19	APB	D	0.3364	0.4420	0.7232	0.4597	0.9405
		ED	0.3830	0.4479	0.6461	0.4017	0.6742
	TA	D	0.2631	0.7033	0.7194	0.6198	0.7498
		ED	0.3811	0.5998	0.5762	0.5446	0.4583
2	APB	D	0.0471	0.0098	0.8671	0.5860	0.8321
		ED	0.4456	0.5051	0.5410	0.4852	0.4076
	TA	D	0.6723	0.6758	0.5796	0.7918	0.7755
		ED	0.6212	0.5787	0.5082	0.6007	0.6257
21	APB	D	1.0000	1.0000	1.0000	0.9714	0.9973
		ED	0.5336	0.6118	0.3364	0.5818	0.5009
	TA	D	0.5650	0.4250	0.6000	0.8900	0.9400
		ED	0.0000	0.0000	0.0000	0.0000	0.0000
3	APB	D	0.9717	0.9217	1.0000	0.9798	0.9869
		ED	0.3702	0.4524	0.3810	0.4935	0.5327
	TA	D	0.5053	0.6325	0.8250	0.6656	0.7125
		ED	0.5343	0.2971	0.5000	0.5700	0.6100
5	APB	D	0.1678	0.8097	0.9123	0.8010	0.9787
		ED	0.3930	0.5710	0.7381	0.6969	0.8672
	TA	D	0.6011	0.6026	0.5801	0.6500	0.6530
		ED	0.5107	0.4725	0.4571	0.3729	0.3214
6	APB	D	0.6754	0.6103	0.7773	0.7920	0.8132
		ED	0.4675	0.5869	0.3779	0.5099	0.5486
	TA	D	0.6568	0.6859	0.5879	0.7004	0.7285
		ED	0.4559	0.5070	0.5257	0.5732	0.6379
7	APB	D	0.6933	0.7351	0.8434	0.8857	0.9010
		ED	0.5842	0.5500	0.6007	0.4226	0.4306
	TA	D	0.8746	0.8216	0.8349	0.9338	0.9426
		ED	0.5404	0.5341	0.6285	0.5031	0.5034

## Footnotes

1 Note that certain CATCH22 features require the interval to have a minimum length of 20.

2 $(x_j)_I^f$ represents the value of feature *f* computed on the subseries corresponding to interval *I*, extracted from the time series $x_j$.
